# Cortical Thinning and Clinical Heterogeneity in Amyotrophic Lateral Sclerosis

**DOI:** 10.1371/journal.pone.0080748

**Published:** 2013-11-20

**Authors:** Domenico Maria Mezzapesa, Eustachio D’Errico, Rosanna Tortelli, Eugenio Distaso, Rosa Cortese, Marianna Tursi, Francesco Federico, Stefano Zoccolella, Giancarlo Logroscino, Franca Dicuonzo, Isabella Laura Simone

**Affiliations:** 1 Neurology Unit, Department of Basic Medical Sciences, Neurosciences and Sense Organs, University of Bari, Bari, Italy; 2 Neurophysiopathology Unit, Department of Basic Medical Sciences, Neurosciences and Sense Organs, University of Bari, Bari, Italy; 3 Neuroradiology Unit, Department of Basic Medical Sciences, Neurosciences and Sense Organs, University of Bari, Bari, Italy; Mayo Clinic, United States of America

## Abstract

Amyotrophic lateral sclerosis (ALS) has heterogeneous clinical features that could be translated into specific patterns of brain atrophy. In the current study we have evaluated the relationship between different clinical expressions of classical ALS and measurements of brain cortical thickness. Cortical thickness analysis was conducted from 3D-MRI using FreeSurfer software in 29 ALS patients and 20 healthy controls. We explored three clinical traits of the disease, subdividing the patients into two groups for each of them: the bulbar or spinal onset, the higher or lower upper motor neuron burden, the faster or slower disease progression. We used both a whole brain vertex-wise analysis and a ROI analysis on primary motor areas. ALS patients showed cortical thinning in bilateral precentral gyrus, bilateral middle frontal gyrus, right superior temporal gyrus and right occipital cortex. ALS patients with higher upper motor neuron burden showed a significant cortical thinning in the right precentral gyrus and in other frontal extra-motor areas, compared to healthy controls. ALS patients with spinal onset showed a significant cortical thinning in the right precentral gyrus and paracentral lobule, compared to healthy controls. ALS patients with faster progressive disease showed a significant cortical thinning in widespread bilateral frontal and temporal areas, including the bilateral precentral gyrus, compared to healthy controls. Focusing on the primary motor areas, the ROI analysis revealed that the mean cortical thickness values were significantly reduced in ALS patients with higher upper motor neuron burden, spinal onset and faster disease progression related to healthy controls. In conclusion, the thickness of primary motor cortex could be a useful surrogate marker of upper motor neuron involvement in ALS; also our results suggest that cortical thinning in motor and non motor areas seem to reflect the clinical heterogeneity of the disease.

## Introduction

Amyotrophic lateral sclerosis (ALS) is a fatal adult onset neurodegenerative disease, characterized by progressive muscle weakness of the bulbar, cervical, thoracic, and lumbosacral regions, reflecting the degeneration of motor neurons in the primary motor cortex, brainstem and spinal cord [1]. In the last few years a large number of researchers have performed magnetic resonance imaging (MRI) analysis of regional volumetric changes in ALS patients, particularly using voxel-based morphometry (VBM), coming to two conclusions. The first is that brain atrophy can be detected not only in motor areas, but also in several non-motor areas, in the frontal, temporal and parietal lobes of both hemispheres [2], [3], confirming the hypothesis that ALS is a brain degenerative disease not confined to the motor system [4]. The second conclusion is that atrophy is highly variable in terms of degree and distribution. Even in the precentral motor area, which is the core of ALS pathology, VBM studies disagree about the presence and degree of volumetric changes [2], [5]. It is possible that these differences are not only attributable to technical issues, but also to intrinsic ALS features.

The clinical expression of ALS is characterized by a large spectrum of heterogeneity [6]. Several phenotypes have been described by various degrees of motor neuron (upper and lower) involvement, site of onset (spinal and bulbar) and cognitive functions, and by different disease progression [7]. This heterogeneity may explain, at least in part, the conflicting results in VBM studies; for instance the prevalence of patients with lower motor neuron signs in a study sample may explain the lack of atrophy in precentral area.

In an effort to find biomarkers of the disease, the role of MRI measures of atrophy would be to translate ALS phenotypic heterogeneity into specific and recognizable neuroradiological patterns. For this purpose, the contribution of VBM has been moderate. VBM, which allows the measurement of grey matter density as a surrogate for cortical volume, is sensitive at group level and not at single patient level [8]. Another MRI analysis recently used in ALS is the measure of brain cortical thickness (CTh). CTh is a property independent from brain volume and is derived from a surface remodelling (namely unfolding and flattening), which allows a good match between homologous brain regions of different subjects [9]. Also CTh can be measured at single subject level. All published studies using CTh measurement agree in showing a reduction in primary motor areas [10]–[13].

The present study was designed to evaluate the relationship between different clinical expressions of classical ALS and brain cortical atrophy, measuring CTh on 3D MRI. First, we explored the pattern of thinning in motor and non-motor brain areas of ALS patients compared to healthy controls (HC). Subsequently, we analysed CTh in three relevant phenotypes of the disease, depicted by the degree of upper motor neuron (UMN) involvement, the site of symptom onset and the rate of disease progression.

## Methods

### Study population

Patients were recruited among outpatients attending the ALS tertiary centre of our Department. All subjects fulfilled revised El Escorial criteria for probable or definite classical ALS, with a sporadic form of the disease [14]. Exclusion criteria were: a) behavioural impairment or change in personality suggestive of fronto-temporal dementia; b) disease onset not precisely attributable to the bulbar or spinal regions; c) inability to perform the imaging study; d) ascertained family history of ALS; e) other major systemic, psychiatric or neurological illness; f) other causes of focal or diffuse brain damage at routine MRI scans.

Each subject underwent a clinical and MRI examination on the same day. The study group totaled 29 ALS patients (14 with probable ALS and 15 with definite ALS; 16 men; mean age ± SD, 58.5±11.0 years). Twenty age-matched HC (9 men; mean age ± SD, 52.4±10.4 years) with no family history of ALS were also studied. All subjects were right-handed. All patients were on treatment with riluzole. Written informed consent was obtained from each participant according to the Declaration of Helsinki and the study was approved by the Local Independent Ethics Committee of the Regional University Hospital Polyclinic of Bari, Italy.

### Clinical assessment

Disease duration was calculated from the date of symptom onset to the date of the MRI. Disease severity was assessed by using the revised ALS Functional Rating Scale (ALSFRS-r) [15]. As an index of disease progression, we calculated the progression rate (PR) by dividing the change of ALSFRS-r by the disease duration: PR =  (48 – ALSFRS-r at “time of examination”)/duration from onset to examination [months] [16]. The site of symptom onset (i.e. bulbar or spinal) was reported by patient history. At the time of the evaluation, patients showed a disability profile dominated by bulbar or spinal signs and symptoms (as estimated by clinical evaluation and ALSFRS-r sub-scores) which were consistent with the site of symptoms at onset. Each patient has been evaluated for a degree of UMN burden, using a clinical scale that takes into account the number, power and site of the UMN involvement in each of the body regions (**Table 1**). Pathologically hyper brisk reflexes, clonus, spastic hypertonia, Babinski and Tromner signs, brisk facial and jaw jerks, and forced yawn were considered as signs of UMN affection. Scores are expressed as a percentage of UMN clinical involvement.

**Table 1:** Scale for clinical evaluation of upper motor neuron burden.

**Deep tendon reflexes:**	
0	absent reflex
1	weak reflex
2	moderate reflex
3	brisk reflex with enlargement of reflexogen area
4	Clonus
right UL ___; left UL ___; right LL___; left LL___	
**Muscle tone**	
0	no spasticity
1	slight spasticity
2	moderate spasticity
3	strong spasticity
4	insuperable spasticity
right UL ___; left UL ___; right LL___; left LL___	
**Babinski sign, Tromner sign, brisk facial and jaw jerks, forced yawn**	
0	absence of these signs
1	one sign present
2	two signs present
3	three signs present
4	all signs present
**Total: ____/36 (___%)**	

UL, Upper Limb; LL, lower limb.

We chose to explore three clinical features of the disease, for each of which two groups have been formed:

- bulbar or spinal (B-ALS and S-ALS respectively) site of onset;
- higher or lower UMN burden (>UMN-ALS and <UMN-ALS respectively), using the 50% of UMN score as cut off to stratify patients (>UMN-ALS, mean score 63.2±10.4; <UMN-ALS, mean score 21.9 ±13.8);
- faster or slower disease progression (FP-ALS and SP-ALS respectively), using the median of PR (0.36) as a cut off to stratify patients.

All subjects underwent a brief neuropsychological examination. After a screening of global cognitive evaluation performed with Mini Mental State Examination [17] to check for the absence of dementia, both patients and controls underwent the FAS test for the phonemic fluency [18], [19], the Stroop color-word test [20], [21] and the symbol digit modality test [22]. Subjects’ scores were expressed as a Z-score (ie, as the number of SDs from the mean) [23].

### MRI acquisition

MRI was performed with a 1.5 Tesla General Electric Signa MR system. Conventional sequences, consisting of dual-echo, in order to obtain a T2 weighted image, and fluid attenuated inversion recovery (FLAIR), were acquired in axial and coronal orientation covering the whole brain. Double fast spin-echo sequence (TR/TE1/TE2, 2100/12/96 ms; matrix, 345×512) and the FLAIR sequence (TR/TE, 8152/102 ms; matrix, 256×256) was performed with 20 slices, 5-mm thickness, 1.0 mm interslice gap, and 24 cm FOV. 3D structural MRI was acquired using a T1-weighted SPGR (Spoiled Gradient Echo) sequence (TR/TE/NEX/flip angle, 26 ms/7 ms/1/50°; FOV, 25 cm; matrix, 256×256; voxel size, 0.97 mm×0.97 mm×1.0 mm).

### CTh measurement

**Figure 1:**
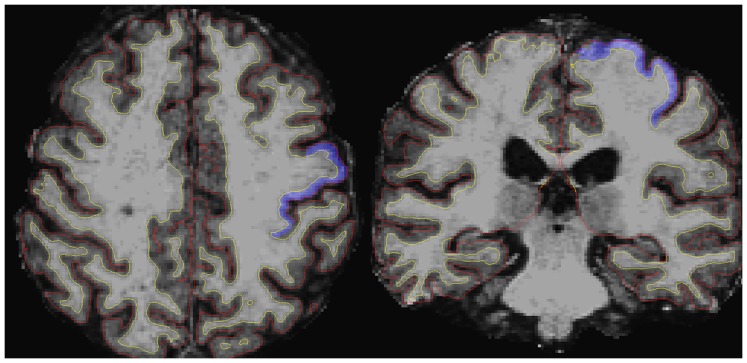
Example of the CTh boundaries of an ALS patients. Axial and coronal T1-weighted images of an ALS patient. White matter surface (yellow line) and pial surface (red line), which are the boundaries of cerebral cortex, have been automatically obtained by Freesurfer. The primary motor area (blu) has been automatically labeled according to the Desikan/Killiany Atlas.

CTh analysis was conducted with the freely available FreeSurfer software package (version 5.1.0), a set of automated tools for reconstruction of the brain’s cortical surface from MRI data (http://surfer.nmr.mgh.harvard.edu) [9]. The processing procedures require 3D T1 weighted MRI data. After skull stripping, segmentation into white and grey matter and tessellation, CTh measurements were obtained by reconstructing representations of the grey/white matter interface and the cortical surface and then by calculating the distance between these surfaces at each point across the cortical mantle [24]. **Figure 1** shows the boundaries of cerebral cortex, i.e. the white matter surface and the pial surface, as defined by the software. By inflating the cortical sulci, three-dimensional inflated and spherical white matter models were created for spatial normalization and vertex-based statistical analysis [25]. The cortex was parcellated in 34 different regions for each hemisphere according to the brain atlas included in Freesurfer (Desikan/Killiany Atlas) and average measures of CTh were produced at each point on the reconstructed surface (vertex) [26].

### Statistical analysis

**Figure 2:**
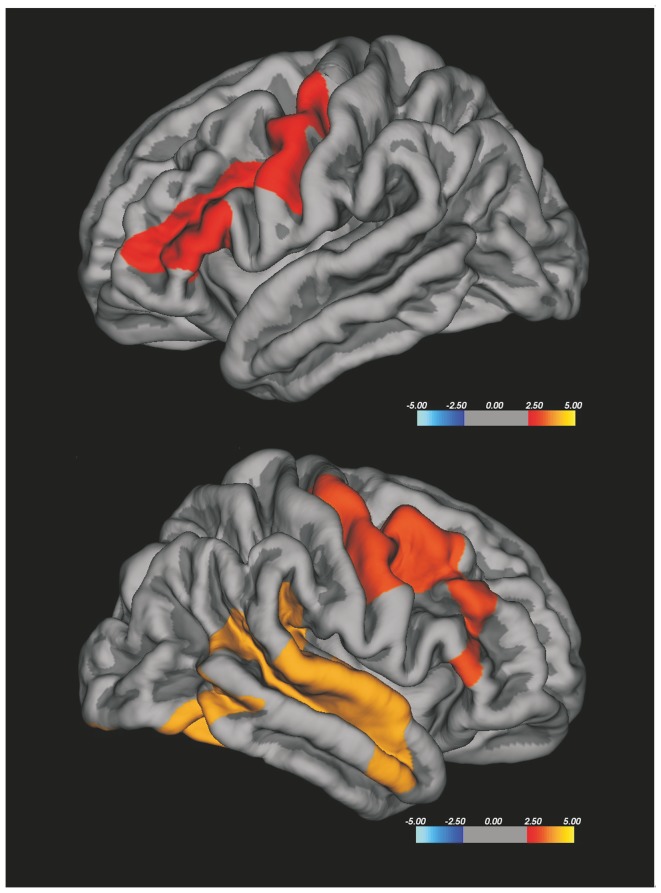
Vertex-wise analysis of CTh in ALS patients related to HC. ALS patients revealed cortical thinning in bilateral precentral cortex, bilateral middle frontal gyrus, right superior temporal gyrus and right occipital cortex. The colour bar scale represents t values.

The group differences of CTh were explored with two types of analysis: a vertex-based analysis and a region of interest (ROI)-based one. The whole brain vertex-wise analysis is a point-by point group comparison of thickness across the cortical surface, without any a priori hypothesis, starting with the average images of each group. This statistical analysis was performed using Qdec, a module of Freesurfer developed to design and execute surface analysis. Age and intracranial volume were introduced in the model as nuisance factors; this was particularly necessary because the controls were slightly younger than the patients. Both group comparison and correlation between variables were performed and results were visualized by overlaying significant cortical areas on the cortical surfaces. To correct for multiple comparisons, after attempting False Discovery Rate methods finding no differences between the clusters, we performed Monte-Carlo cluster-based simulation with 10,000 permutations and we searched for significant clusters with *p* value-level 0.05 [27]. Areas showing significant cortical thinning were superimposed on the template; the colour bar scale represents t values (See **Figures 2** **-** **5**).

**Figure 3:**
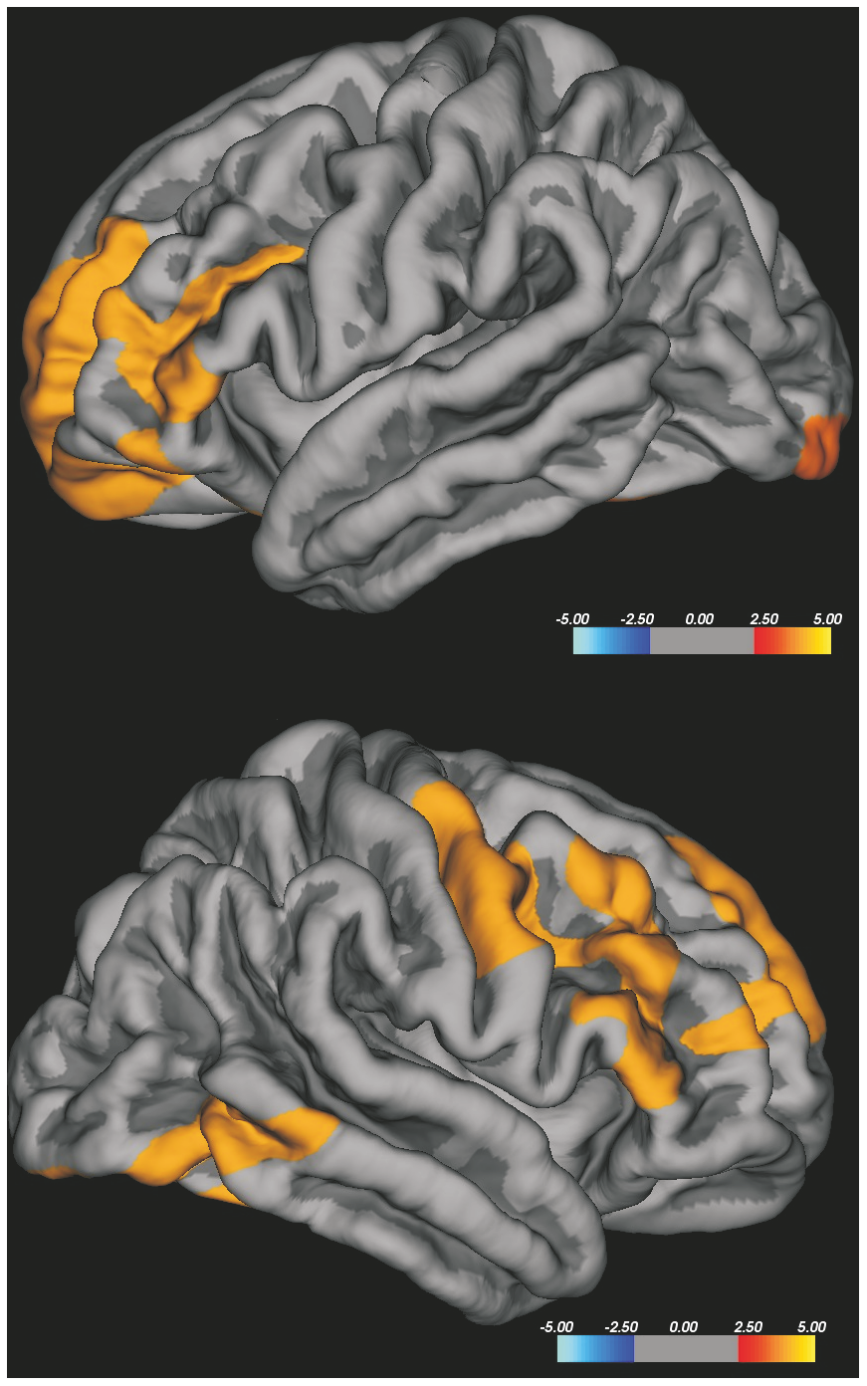
Vertex-wise analysis of CTh in ALS patients with higher UMN burden compared to HC. ALS patients with higher UMN burden showed cortical thinning in the right precentral gyrus, in bilateral superior, middle and inferior frontal gyri, in the right inferior temporal gyrus and in left lateral occipital cortex. The colour bar scale represents t values.

**Figure 4:**
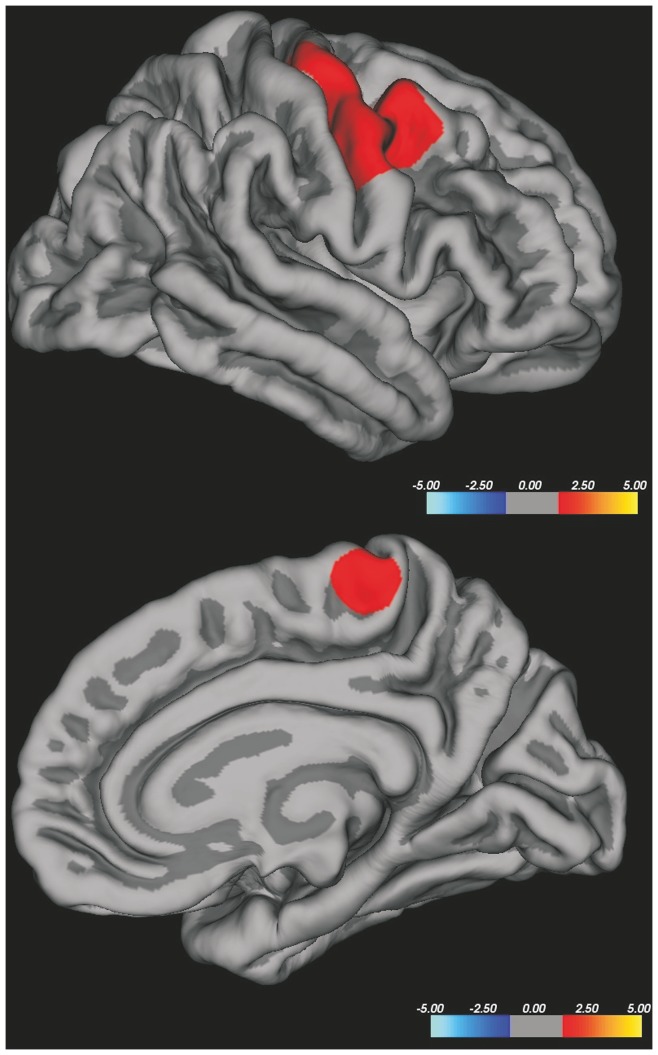
Vertex-wise analysis of CTh in ALS patients with spinal onset related to HC. Spinal onset ALS patients showed a significant thinning in right precentral and paracentral gyri; no differences were revealed in the left hemisphere. The colour bar scale represents t values.

**Figure 5:**
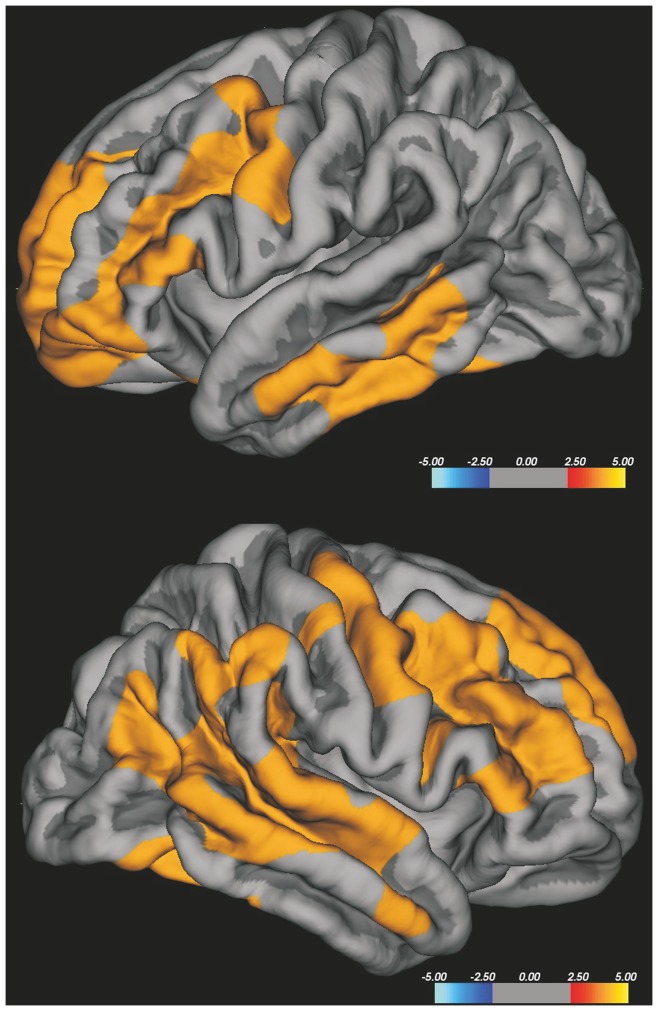
Vertex-wise analysis of CTh in ALS patients with faster progression compared to HC. Faster progressing ALS patients showed relevant thinning in widespread bilateral frontal and temporal areas, including the bilateral precentral gyrus. The colour bar scale represents t values.

Moreover the vertex-analyses were supplemented by a ROI-analysis, using the mean values of CTh in the primary motor areas (i.e. in the precentral gyrus and in the paracentral lobule). In this case the statistical analysis was performed as a simple group comparison.

The differences in frequency of categorical variables were analyzed using the Fisher Exact test. Data comparison between groups was performed using the nonparametric Mann-Whitney *U* test.

## Results

Clinical and demographic features of ALS patients are summarized in **Table 2**. No differences were found in terms of age and gender between ALS and HC.

**Table 2:** Clinical features of ALS patients.

Mean age (years ± SD)	58.5±11.0
**Sex (M/F)**	16/13
**Mean ALSFRS-r (± SD)**	36.59±7.76
**Mean disease duration (months ± SD)**	37.26±39.54
**Progression Rate (± SD)**	0.84±1.07
**>UMN/<UMN burden**	11/18
**B-ALS/S-ALS onset**	10/19

ALS, amyotrophic lateral sclerosis. >UMN, higher upper motor neuron burden. <UMN, lower upper motor neuron burden. B-ALS, bulbar onset of disease. S-ALS, spinal onset of disease.

### CTh comparison between ALS patients and HC

ALS patients showed a slight but not significant decrease of whole hemispheric average CTh compared to HC on both sides **(** **Table 3** **)**. The vertex-wise analysis of CTh, corrected for multiple comparisons, is shown in **Figure 2**. ALS patients revealed cortical thinning in bilateral precentral cortex, bilateral middle frontal gyrus, right superior temporal gyrus and right occipital cortex.

**Table 3:** Mean whole brain and primary motor areas cortical thickness in ALS and HC.

	HC (20)	ALS pts (29)	*% difference*	*p*
**CTh in whole RIGHT hemisphere**	2.43±0.09	2.38±0.08	2.10%	n s
**CTh in whole LEFT hemisphere**	2.43±0.09	2.38±0.09	2.10%	n s
**CTh in RIGHT precentral gyrus**	2.44±0.12	2.37±0.13	2.95%	0.046
**CTh in RIGHT paracentral lobule**	2.32±0.12	2.25±0.13	3.11%	n s
**CTh in LEFT precentral gyrus**	2.48±0.10	2.40±0.14	3.33%	0.036
**CTh in LEFT paracentral lobule**	2.37±0.13	2.27±0.13	4.41%	0.009

CTh, cortical thickness, expressed in millimeters (mean ± SD); ALS, amyotrophic lateral sclerosis; HC, healthy controls; n s, not significant.

The ROI analysis on primary motor areas detected cortical thinning in the bilateral precentral cortex (difference 2.95% on right, p = 0.046; and 3.33% on left, p = 0.036) and in the left paracentral lobule (difference 4.41%, p = 0.009), in ALS patients compared to HC **(** **Table 3** **)**.

### CTh and UMN burden

The clinical characteristics of patients, grouped according to the amount of UMN involvement, are reported in **Table 4**. The two groups significantly differed in terms of progression rate, which was higher (i.e. more rapid progression) in >UMN-ALS patients (p  = 0.002).

**Table 4:** Clinical findings according ALS clinical traits.

	According to >UMN or<UMN			According to bulbar or spinal onset			According to the rate of disease progression		
	>UMN-ALS	<UMN-ALS	*p*	S-ALS	B-ALS	*p*	FP-ALS	SP-ALS	*p*
	pts (11)	pts (18)		pts (19)	pts (10)		pts (14)	pts (15)	
									
Age at evaluation (years)	56.7±6.5	59.3±12.8	n.s.	56.7±10.4	61.9±11.9	n.s.	61.2±9.2	56.0±11.7	n.s.
Sex (M/F)	6/5	10/9	n.s.	13/6	3/7	n.s.	5/9	11/4	0.048
ALSFRS-r	35±7.81	37.56±7.78	n.s.	37.58±6.33	34.70±10.04	n.s.	33.93±8.42	39.07±6.39	n.s.
Disease duration (months)	33.43±48.61	39.60±34.21	n.s.	45.84±45.49	20.96±16.60	n.s.	13±10.79	59.90±43.38	0.001
Progression Rate	1.59±1.43	0.38±0.30	0.002	0.74±1.07	1.03±1.10	n.s.	1.55±1.18	0.18±0.10	-
>UMN-ALS/<UMN-ALS	-	-	-	8/11	3/7	n.s.	7/7	4/11	n.s.
B-ALS/S-ALS onset	3/8	7/11	n.s.	-	-	-	7/7	3/12	n.s.

ALS, amyotrophic lateral sclerosis; >UMN-ALS, higher upper motor neuron burden; <UMN-ALS, lower upper motor neuron burden; S-ALS, spinal onset of disease; B-ALS, bulbar onset of disease; FP, faster disease progression; SP, slower disease progression; n.s., not significant.

ALS patients with >UMN showed a significant decrease in whole hemispheric average CTh compared to HC on both sides. No differences in whole hemispheric average CTh were detected between >UMN-ALS and <UMN-ALS patients, neither between <UMN-ALS patients and HC (**Table 5**).

**Table 5:** Mean whole brain and primary motor areas cortical thickness in ALS subgroups.

CTh	HC (20)	<UMN-ALS	>UMN-ALS	S-ALS	B-ALS	SP-ALS	FP-ALS
		pts (18)	pts (11)	pts (19)	pts (10)	pts (15)	pts (14)
**Whole RIGHT hemisphere**	2.43±0.09	2.39±0.09	2.36±0.08 *	2.38±0.09	2.38±0.07	2.40±0.08	2.35±0.08 *
**Whole LEFT hemisphere**	2.43±0.09	2.39±0.09	2.36±0.09 *	2.38±0.09	2.37±0.09	2.40±0.09	2.35±0.09 *
**RIGHT precentral gyrus**	2.44±0.12	2.39±0.13	2.33±0.11 *	2.35±0.13 *	2.42±0.10	2.40±0.13	2.34±0.12 *
**RIGHT paracentral lobule**	2.32±0.12	2.25±0.14	2.25±0.12	2.22±0.14 *	2.31±0.11	2.32±0.12	2.25±0.14
**LEFT precentral gyrus**	2.48±0.10	2.43±0.13	2.35±0.13 §	2.38±0.14 *	2.43±0.13	2.42±0.13	2.37±0.15 *
**LEFT paracentral lobule**	2.37±0.13	2.29±0.12*	2.24±0.13 *	2.25±0.13 Ω	2.32±0.11	2.29±0.13	2.25±0.13 §

* p<0.05 vs HC, § p≤0.01 vs HC, Ω p<0.005 vs HC.

CTh, cortical thickness, expressed in millimeters (mean ± SD); ALS, amyotrophic lateral sclerosis; HC, Healthy Controls; >UMN-ALS, higher upper motor neuron burden; <UMN-ALS, lower upper motor neuron burden; S-ALS, spinal onset of disease; B-ALS, bulbar onset of disease; FP, faster disease progression; SP, slower disease progression.

The whole brain vertex-wise analysis of CTh, corrected for multiple comparisons, did not show CTh differences between >UMN-ALS and <UMN-ALS patients. Also no differences were found between <UMN-ALS patients and HC, whereas >UMN-ALS patients showed significant CTh reduction in the right precentral gyrus, in bilateral superior, middle and inferior frontal gyri, in the right inferior temporal gyrus and in left lateral occipital cortex in comparison with HC (**Figure 3**).

The ROI analysis on primary motor areas detected cortical thinning in bilateral precentral cortex (difference 4.72% on right, p = 0.02; and 5.53% on left, p = 0.006) and in left paracentral lobule (difference 5.80%, p = 0.012) in >UMN-ALS compared to HC. Also CTh reduction of left paracentral lobule was noticed in <UMN-ALS related to HC (difference 3.49%, p = 0.049). For absolute value see **Table 5**.

### CTh and site of symptom onset

The clinical features of patients, grouped according to spinal or bulbar onset, are shown in **Table 4**. No clinical differences were found between the two subgroups.

No significant differences in whole hemispheric average CTh were detected between S-ALS, B-ALS onset and HC, analyzing each one against another (**Table 5**).

The whole brain vertex-wise analysis of CTh, corrected for multiple comparisons, did not show significant differences between the two groups of patients as well as between B-ALS onset and HC in both hemispheres. Comparing S-ALS onset patients with HC, a significant thinning was found in the right precentral and paracentral gyri (**Figure 4**); no differences were revealed in the left hemisphere.

The ROI analysis on primary motor areas detected cortical thinning in bilateral precentral cortex (difference 3.83% on right, p = 0.022 and 4.20% on left, p = 0.02) and in bilateral paracentral lobule (difference 4.50% on right, p = 0.024 and 5.33% on left, p = 0.004 on left), in S-ALS compared to HC (for absolute value see **Table 5** **)**.

### CTh and disease progression

The clinical features of patients grouped according to the degree of clinical progression are shown in **Table 4**. In relation to sex distribution, a rapid progression was associated with the male sex.

FP-ALS patients showed a significant decrease in whole hemispheric average CTh compared to HC on both sides. No differences on whole hemispheric average CTh were detected between FP-ALS and SP-ALS patients, nor between SP-ALS patients and HC (**Table 5**).

The whole brain vertex-wise analysis of CTh, corrected for multiple comparisons, did not show significant differences between FP-ALS and SP-ALS and between SP-ALS and HC in both hemispheres. FP-ALS compared to HC showed significant differences in widespread bilateral frontal and temporal areas, including the bilateral precentral gyrus (**Figure 5**).

The ROI analysis on primary motor areas detected cortical thinning in bilateral precentral cortex (difference 4.27% on right, p = 0.022 and 4.64% on left, p = 0.024) and in left paracentral lobule (difference 5.33%, p = 0.010), in FP-ALS patients compared to HC (for absolute value see **Table 5** **).**

### CTh and neuropsychological assessment

Patients and HC did not differ in neuropsychological performances for each test. Also we did not find any correlation between test scores and CTh in the whole brain vertex-wise analysis.

## Discussion

In this study we measured the whole brain CTh in patients with ALS in order to identify patterns of thinning related to the disease and to some of its clinical traits. A cortical thinning was evident in the precentral gyrus bilaterally, as well as in non-motor areas, in the frontal lobe bilaterally and in the right temporal lobe, in ALS patients compared to HC. The distinctive aspect of our study is that cortical thinning in both motor and non-motor areas was related to some clinical expression of the disease, such as faster progression, higher UMN burden and spinal symptom onset.

The cortical thinning in primary motor areas of ALS patients was an expected but not an assured finding, because in several VBM studies atrophy of the precentral gyrus was either not found or less prominent than in non-motor areas [2], [3]. In a previous work with VBM, our own group did not find grey matter reduction in these regions [5]. On the contrary, all published studies which use CTh measurement agree in showing a reduction in primary motor areas [10]–[13]. The measure of CTh, which is independent of cortical surface or volume variance, may be more sensitive than VBM in neurodegenerative diseases, as has been demonstrated in Parkinson’s disease and in age-associated grey matter reduction [28], [29]. Another advantage of this technique is that pre-processing steps allow a better inter-subject registration for matching homologous cortical regions, compared to VBM [25]. However, VBM and CTh measurement have not been directly compared in ALS.

An interesting question to be discussed is whether the cortical thinning in primary motor areas can reflect a UMN damage and consequently help the diagnosis of ALS. We adopted a clinical scale that allows us to quantify the UMN involvement. The vertex-wise analysis revealed that the cortical thinning in primary motor areas, although only in the right side, was present in patients with higher UMN and not with lower UMN burden. Focusing the analysis on the primary motor areas with a ROI approach, cortical thinning was also found on the left side. Nevertheless it remains yet to be determined whether the value of the CTh measure in primary motor areas is comparable to that of electromyography for the lower motor neuron. The previous work of Agosta et al. estimated the discriminatory power of CTh between patients and controls at 75% [13]. Similarly, Verstraete et al., using a 3 Tesla MR scanner, detected a cutoff value of CTh in the precentral gyrus with a specificity of 82% and a sensitivity of 84% [12]. Our study was less optimistic regarding the value of CTh as a diagnostic tool because there was considerable overlap of measures between patients and HC, suggesting a lower discriminatory power. Since ALS is characterized by a variable mixture of the UMN and lower motor neuron involvement, the cortical thinning of primary motor areas, which only derives from the UMN damage, must be plugged in a more structured diagnostic protocol.

Another aspect of ALS heterogeneity is related to the body region of onset. In a previous CTh work the relation between the precentral motor cortex thinning and this clinical feature has been searched but not found [12]. A recent VBM study, focusing the analysis only on the primary motor cortex, discovered focal grey matter atrophy in the bilateral bulbar segment of the motor homunculus in patients with bulbar onset compared with those with spinal onset, and vice versa for patients with limb onset [30]. We found that cortical thinning in primary motor areas was present in patients with spinal onset and not in those with bulbar onset. This was bilateral in the ROI analysis, but only in the right side in the vertex-wise analysis, where the distribution fits the cortical representation of the motor homunculus for spinal muscles, with preservation of the bulbar segment. We explain the lack of detection of cortical thinning in ALS with bulbar onset by the low frequency of UMN involvement in the bulbar region, as reported in some studies [31]–[33]; also in our study, the 70% of ALS with bulbar onset had lower UMN burden.

The disease progression has been related with cortical thinning in two previous studies [12], [13]. Agosta et al. found a more severe thinning of the precentral gyrus in patients with a faster rate of disease progression [13]. Similarly, Verstraete et al. found an association between rapidly progressive course and cortical thinning in temporal areas [12]. In our study only ALS patients with faster clinical progression showed cortical thinning in bilateral frontal areas, including the right precentral gyrus and temporal areas. The more aggressive form of ALS seems to have a counterpart in term of cortical thinning that widely extends beyond the primary motor areas. It is important to note that in the group of slower disease progression there were four times more patients with spinal onset, who showed primary motor cortex thinning, than with bulbar onset; therefore a cortical thinning was also expected in this group. This singular relationship between disease progression and the site of onset of ALS is probably due to the ability of CTh measures to identify faster progression only when it depends on higher UMN burden; faster progression in patients with bulbar onset should not be attributed to UMN involvement. In the context of disease progression, a longitudinal analysis would be helpful to reveal the chronological order of cortical thinning. However, Verstraete et al., who have been able to perform it in a subgroup of patients, showed an early involvement of primary motor cortex, but no further increase with the progression of the disease [12].

A peculiar aspect of our study is that subjects performed a neuropsychological examination and no differences were found between patients and HC. This result suggests that a diffuse involvement of cerebral cortex is not necessarily accompanied by cognitive impairment, even if it is of low degree. Recently it has been suggested that extensive extra motor cortical and subcortical involvement, revealed by a multimodal MR approach, may be in association with the *C9orf72* ALS genotype [34]. Unfortunately only in half of our patients the *C9orf72* genotype has been explored, so our current data is insufficient to draw conclusions.

A recurring finding in our study is the right lateralization of cortical thinning found with the vertex-wise analysis, in particular in patients with spinal onset and higher UMN burden. Several VBM studies have reported asymmetrical atrophy of the primary motor areas prevalently on the right side [35], [36]. Also using SPECT for brain perfusion imaging, an asymmetrical decrease of perfusion with prevalence in the right premotor cortex has been reported [37]. Also there is evidence of a possible hemispheric left lateralization of the disease, as suggested by the more frequent symptom onset on the right side, at least with regards to the upper limb [38], [39]. We did not consider the side of onset before the analysis, but a posteriori we noticed a slight prevalence of left side onset (in the spinal region) which may have influenced the results. However, focalizing the analysis on the primary motor areas, using the mean CTh values of the precentral gyrus and the paracentral lobule, the cortical thinning was bilateral in both spinal onset and higher UMN burden patients compared with HC.

Although there are some strengths to this study, including the close relationship between certain phenotypes and determined pattern of cortical thinning, there are some limitations to be considered: a) the relative small number of patients; b) a slightly younger age in some controls than in patients; c) a very long disease duration in some patients; d) a small discrepancy in gender distribution between ALS and HC. Concerning the disease duration, its relation with CTh was indirectly investigated using the progression rate, which depends on both disease duration and the change of ALSFRS-r. Finally, it is possible that gender may have influenced the CTh, but a larger sample is needed in order to explore different patterns in our subgroups.

The search for biomarkers of disease progression is particularly important for both the design of disease-modifying treatment trials and single patient management. The search for radiological biomarkers is still at the beginning [3]. On the contrary several clinical indicators of prognosis have been well depicted from epidemiological studies, such as the bulbar onset of the disease and the prevalence of lower motor neuron signs mainly described in atypical ALS phenotypes [7], [40]. In this context the measurement of CTh could be relevant, even if a correct interpretation of these results must take into account the clinical heterogeneity of ALS and ultimately of the clinical profile of a single patient.
